# Dual-trigger model of CD20 escape: NONO regulation and cryptic splicing induced by transcript overload in pediatric B-ALL

**DOI:** 10.3389/fimmu.2026.1763413

**Published:** 2026-04-01

**Authors:** Moein Kermani, Sebastian Maxeiner, Francesca Alt, Nicole Ziegler, Julian König, Claudia Paret, Jörg Faber

**Affiliations:** 1 University Medical Center of the Johannes Gutenberg-University Mainz, Department of Pediatric Hematology/Oncology, Center for Pediatric and Adolescent Medicine, Mainz, Germany; 2 Theodor Boveri Institute, Julius Maximilians University Würzburg, Biocenter, Würzburg, Germany; 3 University Cancer Center (UCT), University Medical Center of the Johannes Gutenberg University Mainz, Mainz, Germany; 4 German Cancer Consortium (DKTK), Site Frankfurt/Mainz, Germany, German Cancer Research Center (DKFZ), Heidelberg, Germany

**Keywords:** alternative splicing, B-ALL, CD20, cryptic splicing, isoform switching, NONO

## Abstract

**Introduction:**

The B-cell-specific marker CD20 is expressed in various B-cell malignancies, including B-cell acute lymphoblastic leukemia (B-ALL) and serves as a key target for immunotherapies. Reduced or absent CD20 expression has been associated with diminished responses to anti-CD20 antibodies and CD20 directed CAR T-cells. Antigen loss may arise from alternative splicing or transcriptional downregulation of *MS4A1*, the gene coding for CD20, a processes influenced by RNA- and DNA-binding proteins. *NONO*, a non-POU domain-containing octamer-binding protein implicated in several cancers, regulates CD20 surface expression.

**Methods:**

To explore factors associated with heterogeneous CD20 expression, we quantified *MS4A1* transcript levels, profiled *MS4A1* messenger RNA (mRNA) isoforms, and analyzed *NONO* mRNA in pediatric B-ALL samples. In addition, we used an *in vitro* CRISPR/Cas9 knockout model to assess the effects of *NONO* loss on *MS4A1* transcript abundance, isoform distribution, and transcript stability. Plasmid-based overexpression of *MS4A1* was used to examine its effect on splicing.

**Results:**

Loss of *NONO* was associated with increased *MS4A1* transcript levels without detectable changes in isoform distribution or stability, and *NONO* mRNA expression was negatively associated with *MS4A1* mRNA expression in CD20-positive blasts. At diagnosis, two *MS4A1* mRNA isoforms were detected in CD20-positive blasts: The wild-type (WT-CD20) and a shorter variant (D393-CD20), a Δ4–6 multi–exon–skipped isoform that yields a truncated intracellular protein inaccessible to CD20-directed immunotherapies. Although WT-CD20 was the dominant splice isoform, the D393/WT-CD20 ratio correlated positively with overall *MS4A1* transcript abundance. High WT-CD20 transcript abundance further biased splicing toward the D393-CD20 isoform, indicating involvement of cryptic splice sites and potential re-splicing events at the level of mature *MS4A1* mRNA.

**Discussion:**

Together, these findings are consistent with a model in which *NONO* expression and transcript-level dynamics of *MS4A1* are associated with CD20 heterogeneity in pediatric B-ALL. These observations may contribute to understanding variability in CD20 expression and antigen availability in pediatric B-ALL.

## Introduction

B-cell acute lymphoblastic leukemia (B-ALL) is the most common malignancy in children and young adults. B-ALL in children is associated with a very high probability of cure, whereas in adults B-ALL is less common and overall shows significantly poorer long-term outcomes (1). These age-related differences are reflected in typical genetic landscapes: in children, favorable cytogenetic subtypes such as *ETV6-RUNX1* and high hyperdiploidy predominate, whereas adults more frequently harbor high-risk entities such as *BCR-ABL1* and *IKZF1* mutations (2). Although intensive multi-agent chemotherapy regimens cure approximately 85% of pediatric patients, targeted therapies are increasingly being implemented for individuals with high-risk or relapsed/refractory disease. B-ALL cells express several surface antigens, including CD19, CD20, and CD22, which can be targeted by monoclonal antibodies and chimeric antigen receptor (CAR) T-cell therapies. While CD19 remains the primary therapeutic target, interest in CD20-directed strategies has been increasing (3). CD20-positive B-ALL in adults is associated with poorer clinical outcomes, including lower remission rates and higher relapse rates. The addition of rituximab, an anti-CD20 monoclonal antibody, to standard chemotherapy has been shown to reduce relapse incidence and improve survival (4). In the pediatric population 30–50% of ALL patients express CD20 and the expression increases during the induction therapy (5). While some studies report a negative correlation between CD20 expression intensity and outcome, another study found CD20 expression to be correlated with a slightly better prognosis (6, 7). The addition of rituximab to treatment regimens for CD20+ B-ALL pediatric patients has produced encouraging results (8). Current trials are further exploring CD20-targeted strategies in pediatric B-ALL, including CD19/CD20 bispecific CAR-T cells (NCT06503094), trivalent CD19/CD20/CD22 CAR-T cells (NCT05010564), and CD20 CAR-T cells combined with additional antigen targets (NCT05618041). Therefore, analyzing CD20 expression in pediatric B-ALL is important to better understand its clinical relevance and to inform the development of CD20-targeted therapeutic strategies.

CD20 is a hydrophobic transmembrane protein essential for B cell maturation, B cell receptor (BCR) signaling, and cell cycle initiation (9, 10). It is a key therapeutic target in B-cell malignancies (11, 12). Monoclonal antibodies (mAbs) like rituximab eliminate malignant B cells and, when combined with chemotherapy, improve outcomes (13, 14). They act through complement-dependent cytotoxicity, antibody-dependent cellular cytotoxicity, antibody-dependent phagocytosis, and apoptosis (13, 15). However, loss or reduction of CD20 expression may confer therapy resistance and limit efficacy (16, 17).

NONO (non-POU domain-containing octamer-binding protein) is an RNA- and DNA-binding protein that regulates RNA processing, DNA repair, and gene transcription (18–20). It regulates growth-related genes through interactions with enhancers, RNA polymerase II, and transcription factors (20). Dysregulation of NONO contributes to prostate cancer (21), neuroblastoma progression (22), and embryonic heart defects (23). Our earlier work showed that NONO controls CD20 expression at the protein level in B-cell acute lymphoblastic leukemia (B-ALL) (24). In addition, NONO has been shown to play a critical role in early B cell development, particularly at the pro- to pre-B cell transition, and B cell maturation in the spleen. Mice with global or B cell–specific *NONO* deletion exhibited defective B cell maturation, impaired ERK/AKT/NF-κB activation, and increased apoptosis upon BCR stimulation (25).

Alternative CD20 isoforms can influence therapy response. Wild-type CD20 (WT-CD20) is the predominant functional form in normal and malignant B cells, including B-ALL. D393-CD20 lacks the rituximab epitope and is associated with resistance in B-cell malignancies such as follicular lymphoma (FL) and mantle cell lymphoma (MCL). D393-CD20 is expressed in malignant and Epstein-Barr virus (EBV)-transformed B cell lines, but is absent in B lymphocytes from healthy donors (26, 27).

This study investigates molecular mechanisms underlying CD20 loss in pediatric B-ALL. We investigated the association between *NONO* expression and *MS4A1* mRNA levels, alternative splicing patterns, RNA stability, and CD20 expression in patient samples. Additionally, we analyzed D393-CD20 mRNA presence in B-ALL blasts with different CD20 expression at diagnosis and explored cryptic splice sites that may generate isoform diversity and promote immune escape. Understanding CD20 regulation could help develop strategies to overcome resistance in B-ALL.

## Materials and methods

### Sample characteristics and cohort

Twenty-two pediatric B-ALL samples were obtained at diagnosis; the cohort comprised 11 females and 11 males (age range, 0.5–12 years). Immunophenotypes and molecular subtypes are listed in **Table 1**. Surplus diagnostic bone marrow was used in accordance with ethical guidelines. Informed consent was obtained, with approval from the Ethics Committee of Rhineland-Palatinate (No. 2018-13713), in accordance with the Declaration of Helsinki (2013).

**Table 1:** Characteristics of the pediatric leukemic patients.

#	Diagnosis	Molecular subtype	CD19 [%]	CD20 [%]	Blasts [%]	RIN	Sex	Age at diagnosis [year]
1	cALL	Normal karyotype	100	84	95	6.6	Female	3
2	cALL	Deletion in chr. 9	100	96	82	7.0	Male	12
3	cALL	Hyperdiploid (trisomy 21)	100	39	90	9.7	Male	4
4	cALL	Hyperdiploid (trysomy 21)	100	72	92	9.4	Female	5
5	cALL	Monosomie X, *ETV6-RUNX1*	98	7	86	9.0	Female	8
6	cALL	High hyperdiploid	100	28	83	9.1	Male	3
7	cALL	*ETV6-RUNX1*	100	15	90	7.6	Female	4
8	cALL	Hyperdiploid	100	25	72	9.7	Male	6
9	cALL	High hyperdiploid, *IKFZ1* deletion	100	27	80	7.4	Male	4
10	cALL	High hyperdiploidie *WHSC1-PAG1*	100	22	82	6.6	Male	3
11	B-cell precursor ALL	*MLL-AF9*, rearrangement of chr.9	100	32	96	7.4	Male	0,5
12	cALL	Partial gain of chr. 21	100	0	93	N/A	Female	7
13	cALL	High hyperdiploid	99	0	85	N/A	Male	2
14	cALL	*ETV6-RUNX1*	100	0	82	N/A	Male	3
15	cALL	High hyperdiploid	99	1	57	N/A	Male	3
16	cALL	*ETV6-RUNX1*	100	0	85	10	Female	5
17	cALL	Gain of the *ETV6* region without *ETV6* fusion	94	0	90	9.1	Female	1
18	cALL	Normal karyotype	96	0	95	9.5	Female	5
19	cALL	Hyperdiploid with trisomy 21	100	0	92	N/A	Female	3
20	cALL	Normal karyotype	100	0	94	9.7	Male	4
21	cALL	High hyperdiploid	100	81	96	6.2	Female	8
22	Pre-B ALL	*IKZF1-DDC*; *PAX5* und *IKZF1*-deletion; hyperdiploid	97	63	>90	7.8	Male	4

Overview of 22 pediatric B-ALL samples at diagnosis that were analyzed for CD19 and CD20 surface expression, blast percentage, RIN value, sex, and age. Moleculary subtypes are indicated. The quality of the extracted RNA is indicated by the RNA Integrity Number (RIN). “N/A” indicates data not available. High hyperdiploid (>50 chromosomes); cALL, common ALL.

### Cell culture

The human B-ALL cell line 697 was obtained from DSMZ (Braunschweig, Germany) and cultured in RPMI-1640 (Gibco™, Waltham, MA, USA) supplemented with 10% fetal bovine serum (FBS; Gibco™), 1% L-glutamine, and 1% penicillin-streptomycin (both Sigma-Aldrich^®^, Merck KGaA, Darmstadt, Germany). According to DSMZ, the cell line has a near diploid karyotype, and expression of *TCF3-PBX*. Cells were maintained at 37 °C, 5% CO_2_, and subcultured at 70-80% confluency.

### CRISPR/Cas9 knockout

To investigate the role of NONO in CD20 regulation, a knockout (KO) was generated in 697 cells using CRISPR/Cas9. Two target-specific gRNAs were applied in a dual-guide approach. Ribonucleoprotein (RNP) complexes were assembled from crRNA, tracrRNA, and Cas9 nuclease (IDT, Coralville, IA, USA) according to the manufacturer’s protocol and kept on ice until electroporation. For electroporation, 1 × 10^6^ cells were resuspended in electroporation buffer (Bio-Rad) and mixed with RNPs plus electroporation enhancer (IDT) to a final concentration of 4.8 µM RNPs (including enhancer). Cells were electroporated in a GenePulser^®^ cuvette (0.2 cm) using a GenePulser Xcell (Bio-Rad) with the following settings: square wave, 1 pulse, 250 V, 2 ms.

Immediately after electroporation, cells were transferred into prewarmed RPMI-1640 containing 20% FBS and 1% L-glutamine, without antibiotics and cultured for 72 h prior to downstream analyses. Knockout efficiency was verified by qRT-PCR with *NONO*-specific primers (**Supplementary Figure 1**; **Table 2**). The following crRNAs were used: Hs.Cas9.*NONO*.1.AA and Hs.Cas9.*NONO*.1.AB. Alt-R^®^ Cas9 Negative Control crRNA #1 served as a non-targeting control, labeled “Control” in figures. Potential off-target sites were not experimentally assayed; guide selection was based on the manufacturer-provided in silico off-target scoring (IDT).

**Table 2:** Primerlist.

Target/gene	Primer name	Forward primer sequence (5′→ 3′)	Reverse primer sequence (5′→ 3′)
*MS4A1*	WT-CD20 (EX5-6)	TCCGGATCACTCCTGGC	TCAGAGGGATTAGCTGGTTCAC
*MS4A1*	D393-CD20	GAGGATGTCTTCACTGGAACTTG	TTCTTCCTCTTCTTGGATTGGA
*MS4A1*	EX7-8	GCTGGCATCGTTGAGAATGAAT	TTCTTCCTCTTCTTGGATTGGA
*CD19*	CD19	TGCCCCGTCTTATGGAAACC	CTCTTCTTCTGGGCCCACTC
*MS4A1*	CD20 V1	GCCTTGGAGACTCAGATCCT	CCTCTTCCGAGTGACCTTG
*MS4A1*	CD20 V2	GGCCTTGGAGACTCAGAACT	TCATTGAGGGTAGACATGGC
*MS4A1*	CD20 V3	GCCTGGACTACACCACTCAC	TTGCTCTCAAAACTCCTGAGTC
*MS4A1*	CD20 V4	GCCTTGGAGACTCAGATCCT	GTCATTTTGCTCTCAAAACTCCTTAT
*MS4A1*	fl CD20*	ATGACAACACCCAGAAATTC	TTAAGGAGAGCTGTCATTTTCT
*HPRT1*	HPRT	TGACACTGGCAAAACAATGCA	GGTCCTTTTCACCAGCAAGCT
*NONO*	NONO	GGAGGCTCGTGAGAAGCTG	CCGCCGCATCTCTTCTTCAC

Primers marked with an asterisk (*) were described previously (Ref (27)).

### RNA extraction

Total RNA was extracted from frozen cell pellets using the RNeasy Mini Kit (QIAGEN, Hilden, Germany) according to the manufacturer’s instructions. RNA concentration was measured with a NanoDrop (Thermo Fisher Scientific), and integrity was verified on a Bioanalyzer (Bio-Rad) before downstream applications.

### cDNA synthesis

cDNA was synthesized in a Mastercycler Nexus (Eppendorf, Hamburg, Germany) using the PrimeScript™ RT Reagent Kit (TaKaRa Bio Inc., Shiga, Japan) according to the manufacturer’s instructions.

### PCR and quantitative real-time PCR

cDNA samples were amplified by PCR using Taq DNA Polymerase I (Axon Labortechnik, Kaiserslautern, Germany). *CD20* isoforms were visualized with a QIAxcel^®^ DNA High-Resolution Cartridge on a QIAxcel Advanced System (QIAGEN) using the standard protocol. Quantitative real-time PCR was performed using PerfeCTa^®^ SYBR^®^ Green FastMix (Quantabio, Beverly, MA, USA) in LightCycler^®^ 96-well plates on a LightCycler 480 instrument (Roche Diagnostics, Mannheim, Germany). Each reaction contained cDNA corresponding to equal input RNA amounts. All samples were analyzed in technical triplicates. Each biological replicate represents an independently electroporated and cultured cell population processed separately for RNA extraction and downstream analysis. At least three independent biological experiments were performed for cell line–based analyses unless otherwise indicated in the figure legends. Patient sample analyses represent independent biological samples. Relative gene expression was calculated using the 2^−ΔΔCt^ method after normalization to *HPRT1* as a reference gene. *HPRT1* was selected based on stable expression across experimental conditions and patient samples. Primer specificity was confirmed by melting curve analysis and agarose gel electrophoresis where appropriate. No-template controls were included to exclude contamination. Ct values above 35 were considered below reliable detection limits and excluded from analysis. Isoform-specific primers were used to distinguish WT-CD20 and D393-CD20 transcripts (**Table 2**). For isoform ratio calculations, expression values were normalized to the same reference gene prior to ratio determination. Exact sample sizes (n), replicate type, and statistical tests used are specified in the respective figure legends.

### Transfection

Electroporation was performed using the same technical settings (device, cuvette, buffer, enhancer, pulse) as in the CRISPR/Cas9 knockout section. Five groups of 697 cells (1.5 × 10^6^ per group) were prepared: one untreated control and four electroporation groups. For electroporation, cells were resuspended in electroporation buffer supplemented with the IDT enhancer, either without plasmid (mock) or with 10 µg of one of the following pcDNA3.1(+) plasmids (GenScript Biotech, Piscataway, NJ, USA): empty vector, *MS4A1*-pcDNA3.1(+), or EGFP-pcDNA3.1(+). Cells were maintained in the incubator for 48 hours post-transfection before harvesting. Transfection efficiency was assessed by flow cytometry, based on EGFP fluorescence detected in the FITC channel.

### Actinomycin D mRNA stability assay

mRNA stability was analyzed by transcriptional arrest using actinomycin D. *NONO* KO and negative control cells were seeded at 5x10^5^ cells per well into 6-well plates containing supplemented RPMI-1640 and treated with 5 μg/mL actinomycin D (ActD; Sigma-Aldrich^®^, Merck KGaA, Darmstadt, Germany). Cells were harvested at 2, 4, and 6 hours following treatment. Total RNA was isolated using the RNeasy Mini Kit (QIAGEN, Hilden, Germany) according to the manufacturer’s instructions. RNA concentration and purity (A260/A280 and A260/A230 ratios) were assessed using a NanoDrop spectrophotometer (Thermo Fisher Scientific) prior to reverse transcription. qRT-PCR with isoform-specific primers (**Table 2**) quantified WT-CD20 and D393-CD20 mRNA expression. RNA abundance was normalized to the 0-hour control (100%) and log-transformed. Linear regression of log-transformed values against time yielded the decay constant (*k*), from which the mRNA half-life (t_1_/_2_, in hours) was calculated as $t_{\left\{\frac{1}{2}\right\}}=\frac{\text{ln}\left(2\right)}{k}$.

All reactions were performed in technical triplicates. The number of independent biological replicates (*n*) is indicated in the corresponding figure legend.

### Flow cytometry analysis

For flow cytometric analyses, 1×10^5^ cells were resuspended in phosphate-buffered saline (PBS) and stained with 7-aminoactinomycin D (7-AAD) for viability assessment together with fluorochrome-conjugated antibodies against CD45, CD19, and CD20 (all BD Biosciences, San Jose, CA, USA). Antibody concentrations were used according to the manufacturer’s recommendations.

After staining, cells were washed once with PBS and resuspended in PBS containing 1% bovine serum albumin (BSA; Sigma-Aldrich^®^, Merck KGaA, Darmstadt, Germany). Data acquisition was performed on a FACSLyric flow cytometer (BD Biosciences) using BD FACSuite software (version 1.5.0.925).

Data analysis was carried out using FlowJo software (version 10.8.0). Gating was performed sequentially by first excluding debris based on forward- and side-scatter characteristics, followed by singlet discrimination. Viable cells were identified as 7-AAD–negative events. For experiments using cell lines, analyses were performed on the total viable singlet population. For patient-derived samples, leukemic blasts were identified based on CD45 expression and side-scatter properties and further gated as CD19-positive cells.

CD20 expression was quantified both as the percentage of CD20-positive cells and as mean fluorescence intensity (MFI). CD20 positivity thresholds were defined based on unstained and control samples, and gates were applied consistently across experimental conditions within each experiment. MFI values were extracted from the gated live, singlet CD20-stained population and used for quantitative comparisons between experimental conditions.

All flow cytometry experiments were performed using independent biological replicates unless stated otherwise.

### Statistical analysis

Statistical analyses were performed using GraphPad Prism (version 10.6.0). For cell line–based experiments, data represent independent biological replicates, each measured in technical triplicates unless otherwise indicated. Patient samples were treated as independent biological observations. Unpaired two-tailed Student’s *t*-tests, one-sample *t*-tests, or one-way ANOVA were applied as appropriate, as specified in the respective figure legends. Correlation analyses were performed using Pearson or Spearman correlation, depending on data distribution, as indicated in the figure legends.

Given the exploratory and hypothesis-generating nature of this study and the limited cohort size, no correction for multiple testing was applied. Statistical significance was defined as *p* < 0.05 (*), *p* < 0.01 (**), and *p* < 0.001 (***).

## Results

### *NONO* expression negatively correlates with *CD20* expression in leukemic blasts

**Figure 1:**
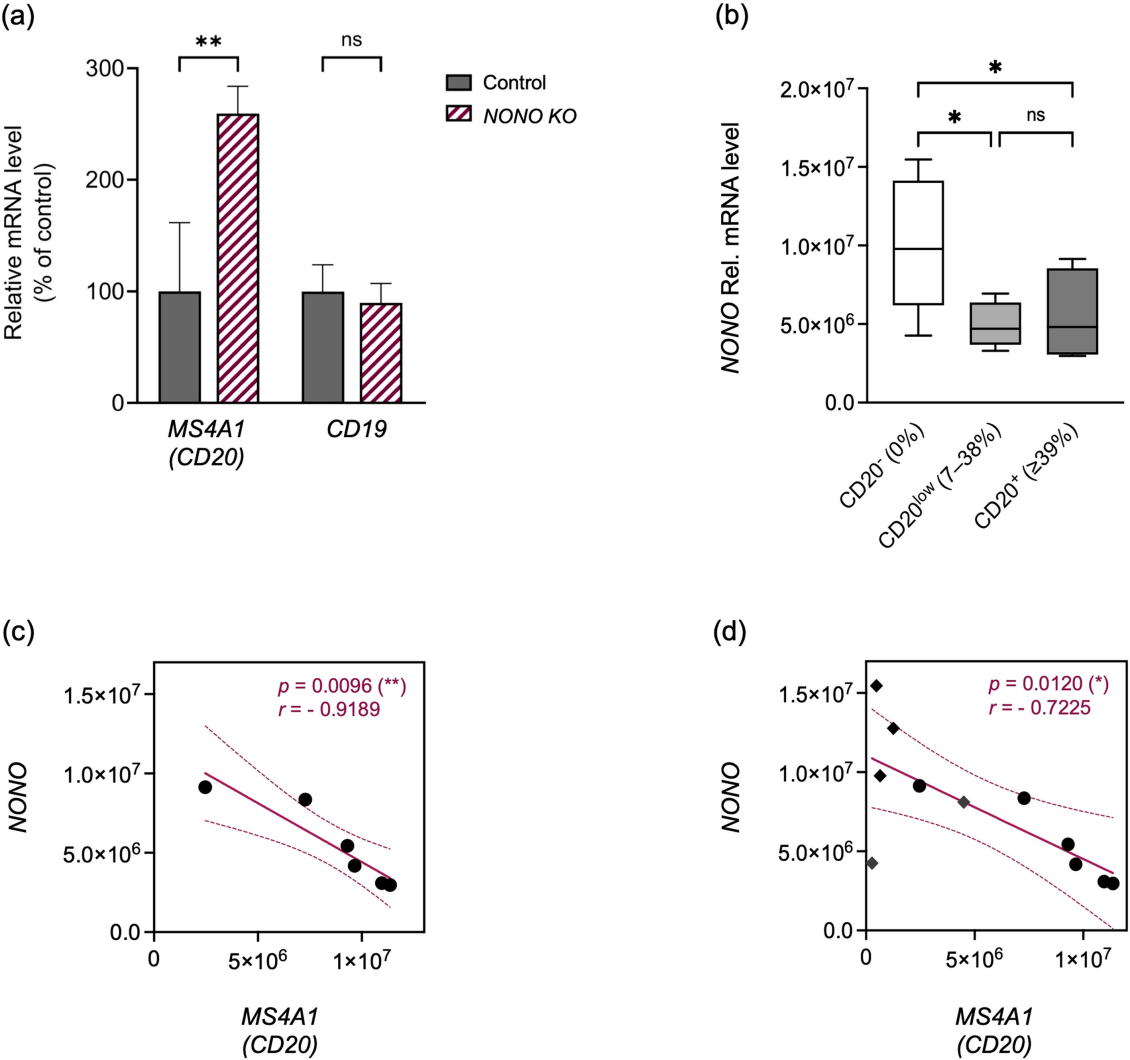
*NONO* expression negatively correlates with CD20 expression in leukemic blasts. **(a)** Relative mRNA levels of *CD19* and *CD20* in non-targeting control and *NONO* knockout (KO) 697 cells, normalized to control values (set as 100%) (mean ± s.d., *n* = 3). Statistical significance was determined by one-way ANOVA. **(b)** Relative mRNA expression of *NONO* in pediatric B-ALL patient samples stratified by surface CD20 expression into three groups: CD20^-^ (0%, *n* = 5), CD20^low^ (7–38%, *n* = 7), and CD20^+^ (≥39%, *n* = 6), analyzed by one-way ANOVA. **(c, d)** Correlation between relative mRNA expression of *NONO* and *MS4A1 (CD20)* in **(c)** CD20^+^ leukemic blasts (*n* = 6) and **(d)** the same CD20^+^ samples together with CD20^–^ blasts analyzed as a mixed cohort (*n* = 11), assessed by two-tailed Pearson correlation with best-fit line; *p* and *r* values shown. Circles indicate CD20^+^ leukemic blasts; diamonds indicate CD20^–^ blasts. Solid lines represent linear regression; dotted lines indicate the 95% confidence interval; *p*< 0.05 (*), *p*< 0.01 (**).

Our previous study showed that *NONO* KO increased CD20-positive cells, while CD19 surface expression remained unchanged (24). Here, we used the same KO and control cells from that study to examine the effect of *NONO* on *CD19* and *MS4A1* mRNA. CD20 is encoded by the *MS4A1* gene, whose mRNA was quantified by qRT-PCR with primers spanning exons 7–8. As shown in **Figures 1a**, *NONO* KO did not alter *CD19* mRNA but significantly upregulated *MS4A1* mRNA. To test whether this inverse relationship exists in patient samples, we analyzed mRNA expression in pediatric B-ALL cases. *NONO* mRNA levels inversely correlated with CD20 surface expression: samples with ≥39% CD20-positive blasts had the lowest *NONO* mRNA, and cases with 7–38% CD20 expression showed reduced *NONO* compared to CD20-negative (0%) cases (**Figure 1b**). Correlation analysis confirmed a negative association between *NONO* and *MS4A1* mRNA in CD20-positive samples (*p* = 0.0096, *r* = −0.9189; **Figure 1c**). Including both CD20-positive and negative cases confirmed the inverse association, with the highest *NONO* concentration in CD20 negative samples, although the correlation was primarily driven by the CD20-positive subgroup (*p* = 0.0120, *r* = −0.7225; **Figure 1d**).

Together, these results show that *NONO* KO increases *MS4A1* mRNA *in vitro* and that reduced endogenous *NONO* is associated with higher CD20 in pediatric B-ALL samples.

### Knockout of *NONO* increases all 5’ UTR splice variants

**Figure 2:**
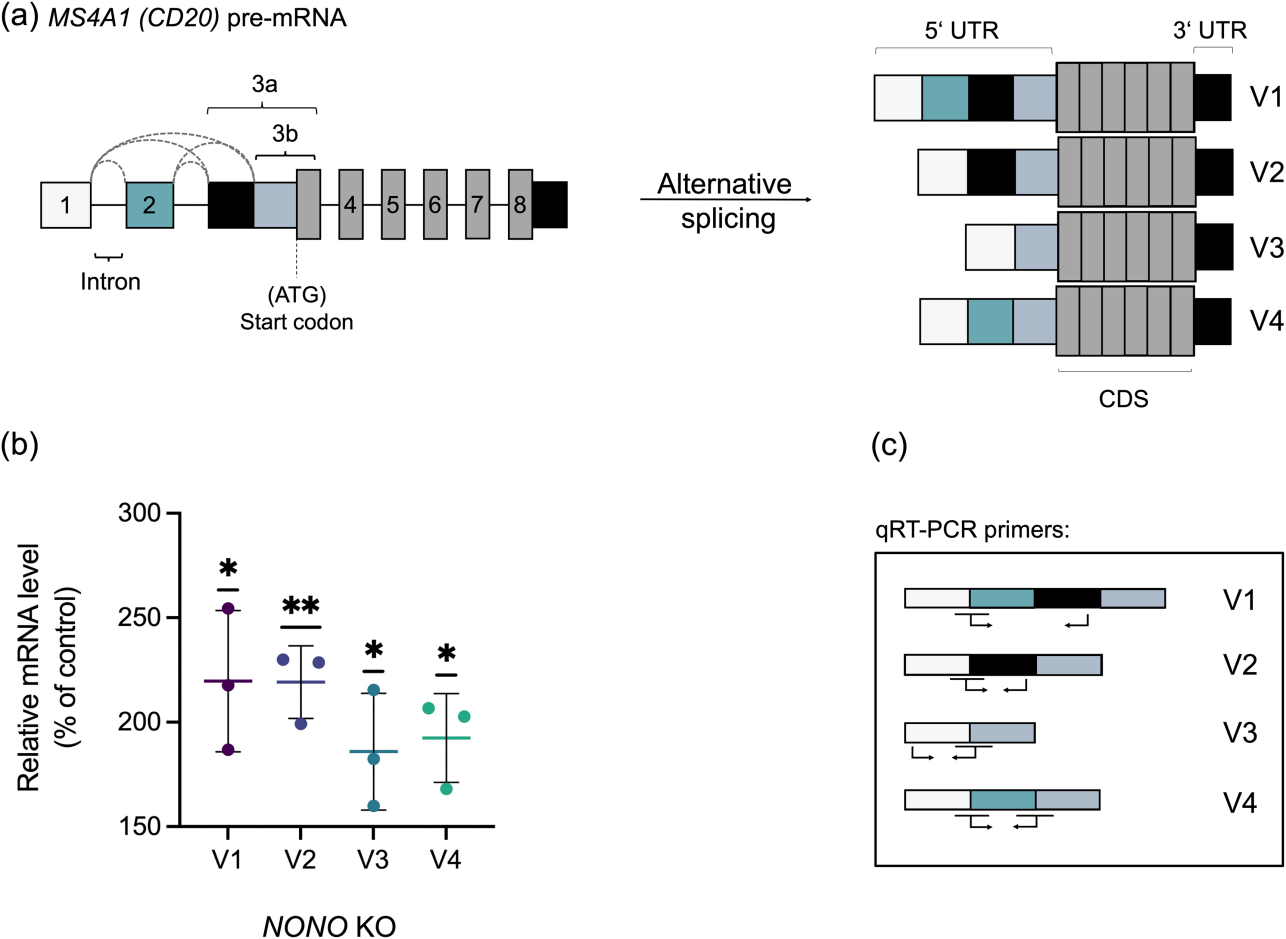
Knockout of *NONO* increases all 5’ UTR splice variants. **(a)** Schematic of *MS4A1 (CD20)* pre-mRNA and 5′ UTR alternative splicing. Exons 1–2 together with the 5′ portion of exon 3 constitute the 5′ UTR, whereas the remaining part of exon 3 and exons 4–8 encode the coding sequence (CDS) (adapted from Ang et al. (30)). **(b)** qRT–PCR analysis of four distinct 5′ UTR splice variants (V1–V4) in *NONO* knockout (KO) 697 cells. Data are presented as mean ± s.d., *n* = 3. One-sample *t*-tests were performed to compare the mean of each variant to the value of 100%, corresponding to the control; *p*< 0.05(*); *p*< 0.01(**). **(c)** Schematic representation of primer binding sites used to specifically quantify four transcript variants (V1–V4). Arrows indicate forward and reverse primers.

*MS4A1* undergoes alternative splicing in its 5′ untranslated region (5′ UTR) (28–30). Although this does not alter the coding sequence (CDS), it modulates CD20 protein levels (30). Ang et al. identified four *MS4A1* variants (V1–V4), differing in exon composition and 5′ UTR structure (**Figure 2a**) (30). V1 and V2 contain extended 5′ UTRs with increased transcript stability but reduced ribosome recruitment, whereas V3 and V4 possess shorter 5′ UTRs that promote more efficient translation. Accordingly, CD20 protein levels correlate positively with the abundance of V3 and, to a lesser extent, V4. These findings highlight the role of 5′ UTR alternative splicing in modulating CD20 expression, with potential implications for response to CD20-targeted immunotherapies (30). To assess NONO*’s* role, we quantified all four variants. qRT-PCR showed that *NONO* KO in 697 cells significantly upregulated V1–V4 (**Figures 2b, c**). *NONO* KO led to an overall increase in *MS4A1* transcript abundance without altering the relative distribution of 5′ UTR isoforms.

### Leukemic blasts express a CD20 isoform lacking the therapeutic epitope at diagnosis

**Figure 3:**
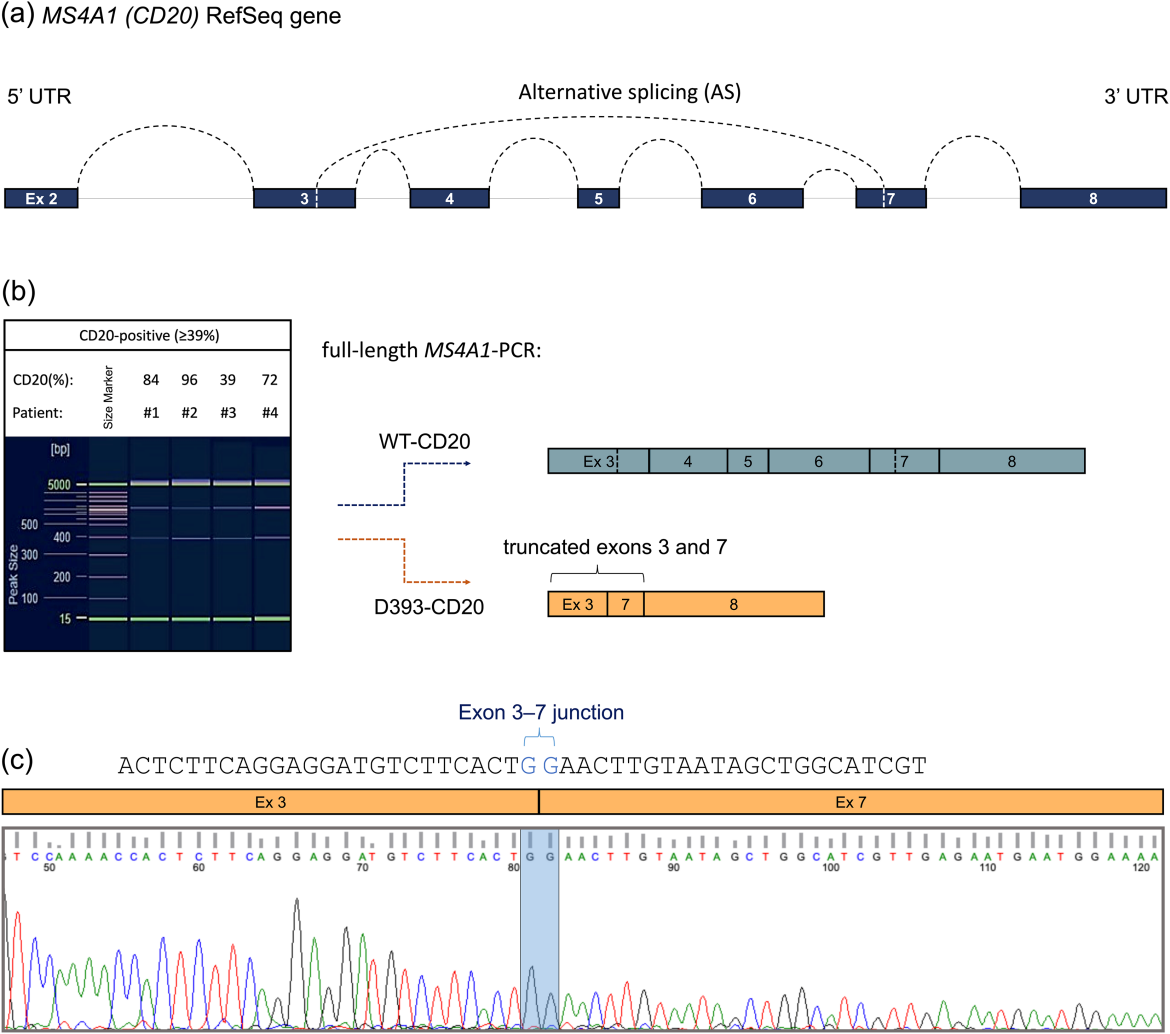
Leukemic blasts express a CD20 isoform lacking the therapeutic epitope at diagnosis. **(a)** Schematic representation of the *MS4A1* gene and its splice patterns across exons 2–8. **(b)** Identification of two CD20 isoforms, WT-CD20 and D393-CD20, by PCR and visualization using capillary gel electrophoresis in CD20-positive leukemia patients (≥39% surface protein expression, *n* = 4). **(c)** Visualization of the D393-CD20 variant using Sanger sequencing electropherograms. The exon–exon junction (exon 3–7) is highlighted in blue.

Besides NONO-mediated regulation, alternative splicing critically modulates CD20 expression. As illustrated in **Figures 3a**, the *MS4A1* gene comprises eight exons that can undergo alternative splicing to generate distinct transcript variants. CD20 isoform variations were analyzed by PCR using a single primer pair spanning exons 3–8 of the *MS4A1* CDS in four pediatric B-ALL samples that exhibited ≥39% CD20 positivity. The WT-CD20 band measured approximately 894 bp (**Figure 3b**). An additional band of ∼ 393 bp was also detected (**Figure 3b**). Sanger sequencing showed that this shorter isoform lacks exons 4–6 and contains truncated forms of exons 3 and 7 (**Figure 3c**), corresponding to the in-frame D393-CD20 isoform, which encodes an intracellular protein that is inaccessible to CD20-directed immunotherapies (26).

### Co-expression of D393-CD20 and WT-CD20 mRNA correlates with surface CD20 levels in leukemic blasts

**Figure 4:**
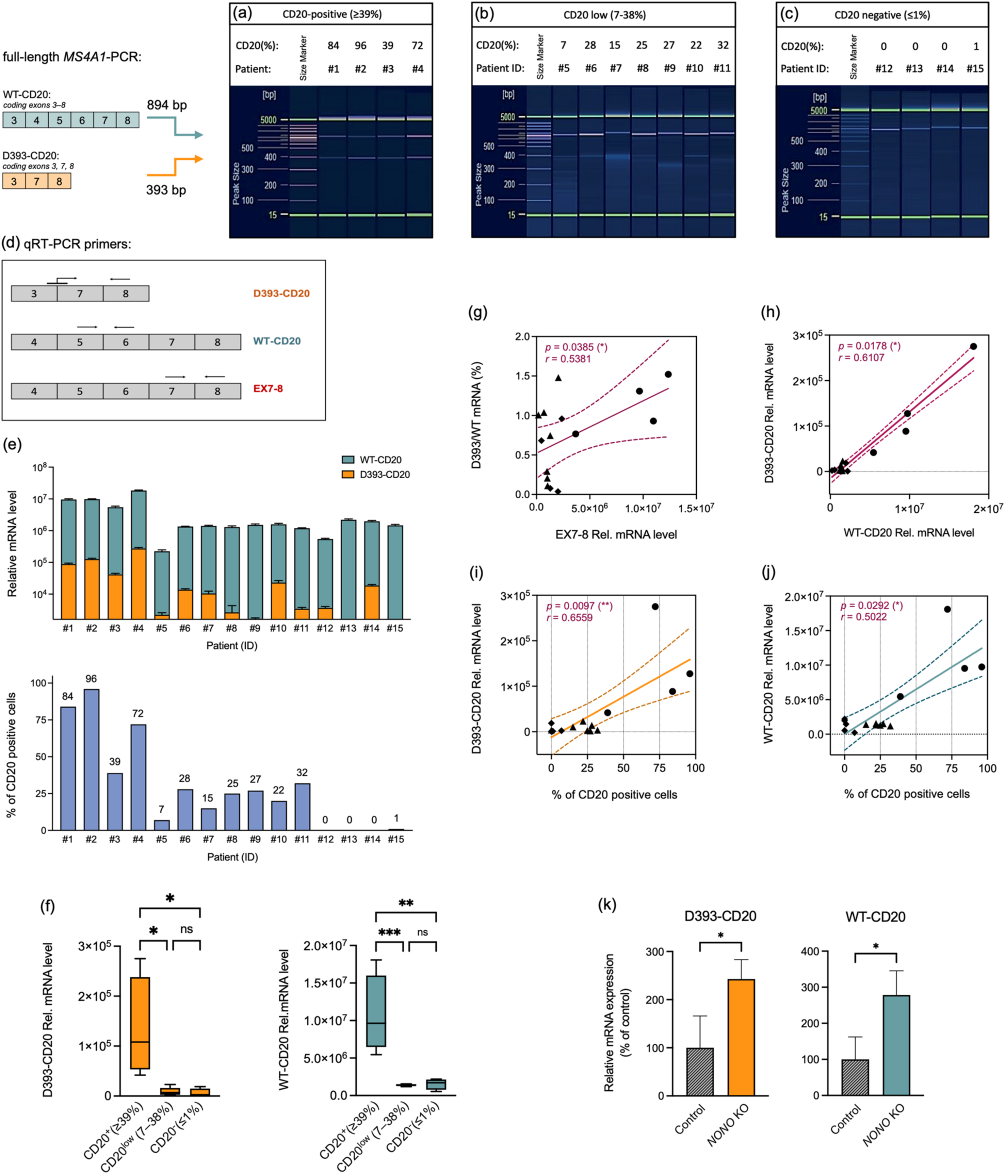
Co-expression of D393-CD20 and WT-CD20 mRNA correlates with surface CD20 levels in leukemic blasts. (a–c) PCR amplification and capillary electrophoresis of WT-CD20 and D393-CD20 isoforms in **(a)** CD20^+^ (≥39%, *n* = 4), **(b)** CD20^low^ (7-38%, *n* = 7), and **(c)** CD20^-^ (≤1%, *n* = 4) leukemia patients. **(d)** Schematic representation of isoform-specific qRT–PCR primers mapped to the *MS4A1* CDS. Primers spanning exons 3–7–8 selectively detect the alternative isoform D393-CD20, whereas primers spanning exons 5–6 amplify the WT-CD20 transcript. Primers spanning exons 7–8 serve as a common control for total *MS4A1* mRNA expression; Arrows indicate forward and reverse primers. **(e)** Relative mRNA abundance of WT- and D393-CD20 transcripts across individual patients by qRT–PCR (top) and corresponding % of CD20 positive cells by flow cytometry (bottom). **(f)** Relative mRNA expression of WT- and D393-CD20 stratified by surface CD20 groups. Significance assessed by one-way ANOVA (ns, not significant; *p*< 0.05 (*), *p*< 0.01 (**), *p*< 0.001 (***)). (g–j) Correlation analyses in pediatric B-ALL blasts (*n* = 15). **(g)** D393/WT-CD20 mRNA ratio (%) *vs* total *MS4A1* (EX7–8) mRNA, **(h)** D393-CD20 mRNA *vs* WT-CD20 mRNA, **(i)** D393-CD20 mRNA *vs* CD20 protein (%), and **(j)** WT-CD20 mRNA *vs* CD20 protein (%). Symbols indicate patient subgroups (circles, CD20^+^; triangles, CD20^low^; diamonds, CD20^-^). Solid lines represent linear regression and dotted lines the 95% confidence interval; Spearman correlation coefficients (*r*) and *p* values are indicated. **(k)** Histogram of WT- and D393-CD20 transcript levels in non-targeting control and *NONO* knockout 697 cell line (mean ± s.d., *n* = 3); statistical testing by unpaired two-tailed *t*-test; *p*< 0.05 (*).

B-ALL blasts were stratified by CD20 surface expression: CD20-positive (≥39%; **Figure 4a**), CD20^low^ (7–38%; **Figure 4b**), and CD20-negative (≤1%; **Figure 4c**). To assess WT-CD20 and D393-CD20 expression, the full-length *MS4A1* CDS (exons 3–8) was PCR-amplified. D393-CD20 was strongly detected in samples with ≥39% CD20 positivity (**Figure 4a**) but was absent or minimal in CD20^low^ (**Figure 4b**) and CD20-negative samples (**Figure 4c**).

qRT–PCR analysis demonstrated interpatient variability in the abundance of WT- and D393-CD20 transcripts, with patients #1–4 with CD20^+^ ≥39% showing relatively elevated levels of both isoforms compared with the other patients (**Figures 4e**, top). D393-CD20 levels were markedly upregulated in CD20^+^ (≥39%) patients compared with CD20^low^ (7–38%) and CD20^-^ (≤1%) cases, whereas no significant difference was observed between CD20^low^ and CD20^-^ groups (**Figure 4f**). Although WT-CD20 represented the predominant transcript across all patients, the D393/WT-CD20 mRNA ratio (%) correlated positively with total *MS4A1* transcript abundance (EX7–8) (*p* = 0.0385, *r* = 0.5381; **Figure 4g**). Moreover, D393-CD20 mRNA correlated positively with WT-CD20 mRNA (*p* = 0.0178, *r* = 0.6107; **Figure 4h**) and with CD20 surface protein (%) (*p* = 0.0097, *r* = 0.6559; **Figure 4i**). **Figure 4j** shows that WT-CD20 transcriptional output is efficiently reflected at the protein level. WT-CD20 mRNA levels correlate positively and approximately linearly with CD20 surface expression (%) (*p* = 0.0292, *r* = 0.5022; **Figure 4j**). Correlation analyses were based on 15 pediatric B-ALL patient samples (*n* = 15).

D393-CD20 levels were markedly upregulated in CD20^+^ samples (≥39%). We therefore examined whether high CD20 expression was associated with a specific molecular subtype (**Table 3**). High expression was observed in six out of 22 samples (27%). Among these, one had a normal karyotype, one showed a deletion on chromosome 9, and six were aneuploid (including three hyperdiploid and one high-hyperdiploid cases). All four *ETV6-RUNX1* samples exhibited low CD20 expression, however, due to the small sample size, this difference does not reach statistical significance. Hyperdiploid karyotypes (any form) were observed in 11 of 22 cases, of which 4 (36%) showed CD20 high expression; however, the association between hyperdiploidy and CD20 expression did not reach statistical significance. Of the six samples with high CD20 expression, three were from female patients and three from male patients, with ages ranging from 3 to 12 years. Overall, no significant associations were observed; however, the cohort size was too small to draw definitive conclusions regarding potential correlations. To explore NONO’s role in isoform regulation, we compared D393- and WT-CD20 mRNA levels in the *NONO* KO 697 cell line. All CD20 transcripts increased significantly and proportionally, with no preferential upregulation of a specific isoform (**Figure 4k**).

**Table 3:** Association between CD20 high expression and clinical variables.

Variable	Category	CD20 high (*n*=6)	CD20 low (*n*=16)	Statistical test	*p*-value
Sex	Male	3	9	Fisher’s exact	1.00
	Female	3	7		
Subtype	Hyperdiploid	4	7	Fisher’s exact	0.36
	Other subtypes	2	9		
Subtype	*ETV6-RUNX1*	0	4	Fisher’s exact	0.28
	Other subtypes	6	12		
Age at diagnosis (years)	Mean ± SD	6.0 ± 3.4	4.1 ± 2.9	Welch t-test	0.27

CD20 high expression is defined as (≥39%).

### NONO does not regulate the stability of CD20 splice isoforms in 697 cells

**Figure 5:**
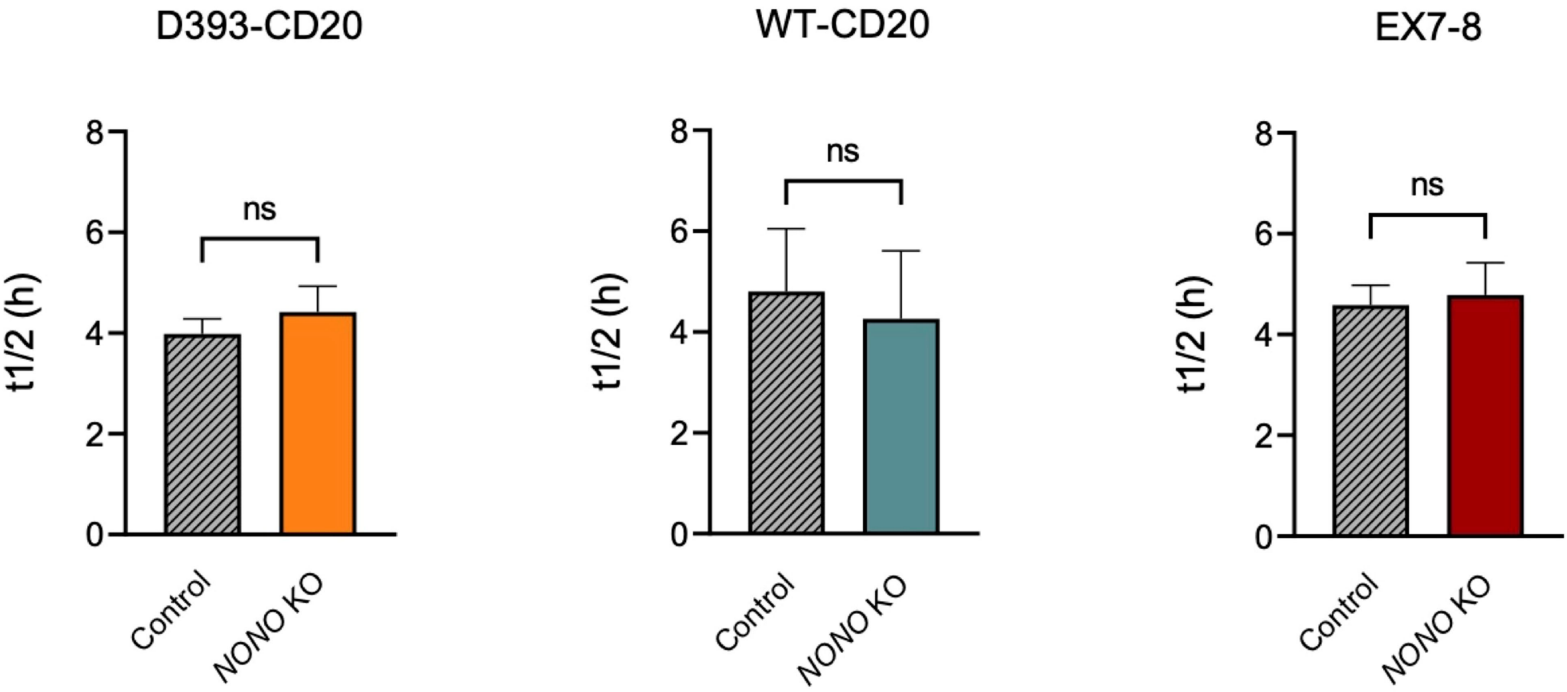
NONO does not regulate the stability of CD20 splice isoforms in 697 cells. mRNA half-lives (t_1_/_2_, in hours) were measured after actinomycin D (ActD) treatment for D393-CD20, WT-CD20 (exon 5-6), and a common region present in both isoforms (EX7-8), in non-targeting control and *NONO* KO cells. No significant differences in transcript stability were observed across conditions (unpaired *t*-test, ns). Data represent mean ± s.d. from *n* = 4 biological replicates.

To test whether NONO affects CD20 transcript stability, we performed an actinomycin D (ActD) mRNA stability assay after *NONO* KO in 697 cells. RNA was isolated at 2, 4, and 6 hours following treatment; untreated samples at 0 hours served as baseline control. qRT-PCR was performed, and isoform half-lives were compared to controls across four independent experiments using an unpaired Student’s *t*-test. After blocking transcription with ActD, *MS4A1* mRNA decayed with similar half-lives in *NONO* KO and non-targeting control cells for each isoform (**Figure 5**). Thus, although NONO suppresses overall CD20 transcript levels, it does not alter degradation kinetics. WT- and D393-CD20 half-lives remained statistically unchanged after *NONO* KO, indicating NONO does not regulate CD20 via mRNA stability.

### WT-CD20 overexpression promotes D393-CD20 splice isoform enrichment in 697 cells

**Figure 6:**
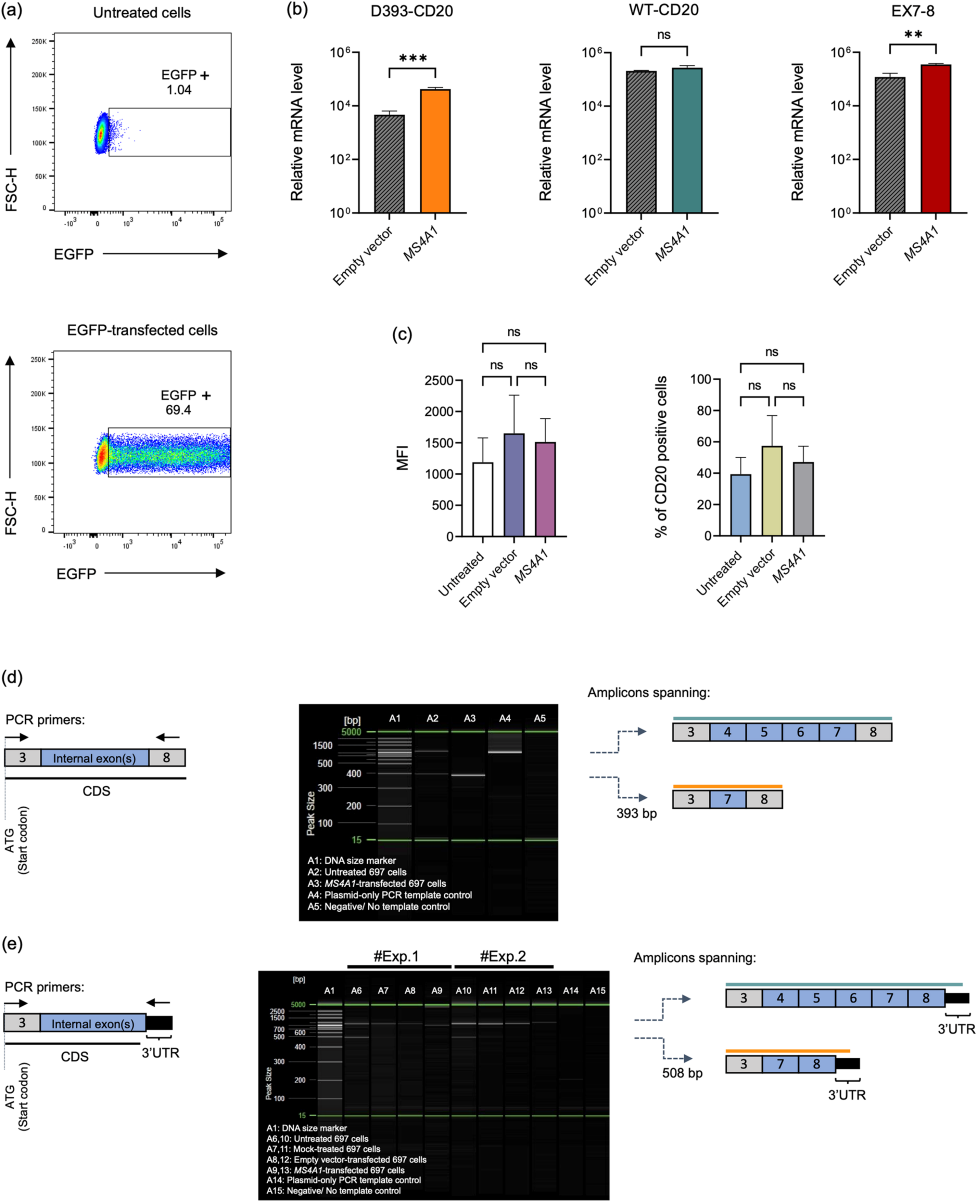
WT-CD20 overexpression promotes D393-CD20 splice isoform enrichment in 697 cells. **(a)** Flow cytometry analysis of EGFP-transfected cells. Dot plots display EGFP fluorescence measured in the FITC channel plotted against FSC-H, shown for untreated (top) and EGFP-transfected (bottom) 697 cells. **(b)** qRT–PCR analysis of *CD20* isoforms in 697 cells transfected with either empty vector (grey hatched) or *MS4A1*-pcDNA3.1(+) (solid bars). Data represent mean ± s.d., *n* = 3. Statistical significance was determined by an unpaired two-tailed *t*-test; ns, not significant; *p*< 0.01 (**) and *p*< 0.001 (***). **(c)** Flow cytometry analysis of surface CD20 after WT-CD20 transfection. Mean fluorescence intensity (MFI) of CD20 surface staining (left) and % of CD20 positive cells (right) were quantified in untreated, empty vector, and *MS4A1*-transfected 697 cells. Data are shown as mean ± SD from *n* = 3 biological replicates. One-way ANOVA confirmed the absence of significant differences across conditions. **(d)** Identification of two *CD20* isoforms, WT-CD20 and D393-CD20, was achieved by PCR using a single primer pair spanning exons 3–8 of the *MS4A1* CDS. The resulting PCR products were separated by capillary gel electrophoresis on the QIAxcel Advanced System, allowing a clear distinction between the full-length WT-CD20 (∼894 bp) and the truncated D393-CD20 isoform (~393 bp). Lane A1: DNA size marker; A2: Untreated 697 cells; A3: *MS4A1*-transfected 697 cells (*CD20* overexpression); A4: Plasmid-only PCR template control; A5: Negative/No template control. **(e)** Capillary gel electrophoresis of PCR products amplified with primers spanning exon 3 to the endogenous 3′ UTR of CD20. The full-length WT-CD20 (∼1,009 bp) band is detected in all samples (A6 to A13). Lane A1: DNA size marker; A6, 10, Untreated 697 cells; A7, 11, Mock-treated 697 cells; A8, 12, Empty vector-transfected 697 cells; A9, 13, *MS4A1*-transfected 697 cells; A14, Plasmid-only PCR template control; A15, Negative/No template control.

The observed co-expression pattern of the D393-CD20 splice isoform and WT-CD20, together with their positive association with the proportion of CD20-positive cells, prompted us to examine whether excessive levels of the WT-CD20 transcript might shift isoform distribution toward the shorter D393-CD20 variant. To address this, we performed an overexpression experiment in which 697 cells were electroporated with a pcDNA3.1(+) vector carrying the full-length *MS4A1* coding sequence. Flow cytometry with EGFP-transfected cells confirmed the efficiency of the transfection (**Figure 6a**).

qRT-PCR using exon 7–8 spanning primers revealed a pronounced increase in total *MS4A1* in CD20-transfected cells compared with empty vector control. Quantification of individual splice variants revealed significant enrichment of D393-CD20, whereas the WT-CD20 transcript remained statistically unchanged (**Figure 6b**). Accordingly, the CD20 surface protein level of *MS4A1*-transfected cells did not differ from untreated or empty vector controls (**Figure 6c**).

PCR results confirmed the enrichment of D393-CD20 under overexpression conditions, as shown in **Figure 6d**. Primers spanning exons 3–8 of the *MS4A1* CDS, followed by capillary gel electrophoresis, detected the full-length WT-CD20 (∼894 bp) and D393-CD20 (∼393 bp) isoform. In *MS4A1*-transfected cells, D393-CD20 was strongly amplified (lane A3; **Supplementary Figure 2**) compared to the untreated cells (lane A2). Sequencing of the short isoform confirmed its identity and ruled out PCR artifacts. The plasmid-only PCR template control (lane A4) yielded a single WT-CD20 band, confirming assay specificity, and no band was detected in the negative control (lane A5).

To assess whether the upregulated D393-CD20 isoform originates from the transfected plasmid or the endogenous locus, we applied a primer pair spanning exon 3 to the endogenous 3′ UTR (**Figure 6e**). This design selectively amplifies endogenous transcripts, as the *MS4A1*-pcDNA3.1(+) plasmid lacks the native 3′ UTR. A ∼508 bp product corresponding to endogenous D393-CD20 was detected in untreated 697 cells (**Figure 6e**; lanes A6, A10), whereas the prominent D393-CD20 band observed with CDS-targeted PCR (**Figure 6d**; lane A3) was absent in *MS4A1*-transfected 697 cells (**Figure 6e**; lanes A9, A13). Taken together, despite only a modest rise in WT-CD20 alongside increased total *MS4A1*, the D393-CD20 isoform was markedly enriched. Across our isoform-resolved assays, the predominant D393-CD20 signal aligns with plasmid-derived transcripts rather than endogenous expression.

## Discussion

B-ALL is characterized by the abnormal proliferation of blasts, which often exhibit aberrant expression of surface markers, including CD20 (31, 32). In contrast to mature B cells, certain B-ALL subtypes express lower levels of CD20 on leukemic blasts, which may have therapeutic implications (31, 33). Patients with CD20-positive B-ALL may benefit from monoclonal antibody therapy in combination with standard chemotherapy, as several studies have reported improved outcomes (34). Consequently, it is important to identify the regulatory mechanisms driving CD20 expression in B-ALL to optimize therapeutic strategies and predict the outcome of targeted treatments.

RNA-binding proteins (RBPs) can govern RNA in terms of splicing, stability, and translation through sequence-specific interactions, typically within UTRs or intronic regions. For instance, PTBP1 is a splicing regulator whose binding to intronic regions of pre-mRNA is crucial for its accurate recognition and removal. In B-ALL blast cells, downregulation of PTBP1 leads to aberrant retention of intron 2 within the *CD19* mRNA, resulting in decreased levels of functional CD19 protein at the cell surface (24, 35). Consequently, PTBP1 helps to stabilize CD19 expression, and its loss can promote the generation of aberrant CD19 isoforms that lack therapeutic target epitopes (24, 35).

In this study, we investigated the role of NONO in regulating *MS4A1* mRNA expression to elucidate potential mechanisms that may account for the previously observed changes in CD20 surface expression after *NONO* KO (24).

The findings of this study provide three complementary observations consistent with an association between NONO expression and CD20 regulation. First, analysis of leukemic blast samples revealed that *NONO* mRNA expression was significantly elevated in CD20-negative blasts and declined with increasing CD20 expression, indicating a stable inverse relationship at the mRNA level. Second, genetic manipulation of *NONO in vitro* using the 697 cell line demonstrated that *NONO* depletion was sufficient to significantly increase *MS4A1* mRNA, whereas *CD19* expression, a stable marker of B cell lineage, remained unaffected. This suggests that NONO expression is inversely associated with CD20 transcript levels. Third, our results revealed that NONO does not influence alternative splicing or mRNA stability of CD20 isoforms. The transcriptional induction of *MS4A1* upon *NONO* loss, together with prior evidence that NONO can act as a chromatin-associated regulator in other contexts (e.g., γ-globin silencing) (36), supports a model in which NONO contributes to transcriptional restraint of *MS4A1*. However, direct chromatin-based evidence at the *MS4A1* locus was not assessed in this study. Thus, the observed CD20 upregulation is compatible with effects at the transcriptional level; however, whether this reflects direct chromatin-associated regulation by NONO or indirect mechanisms cannot be determined based on the current data. Notably, NONO is predominantly described as an RNA-binding and paraspeckle-associated protein, and its regulatory effects on gene expression are frequently mediated through post-transcriptional or indirect mechanisms. Therefore, the observed increase in *MS4A1* transcripts does not necessarily imply direct promoter binding. Nevertheless, a contribution of NONO to post-transcriptional processes such as mRNA export and translation efficiency cannot be definitively excluded.

It should be noted that the correlation analyses presented between *NONO* and *MS4A1* mRNA expression in both cohorts are based on a limited number of patients (*n* = 6 for **Figure 1**.c and *n* = 11 for **Figure 1d**) and therefore have limited statistical power and should be interpreted with caution and in an exploratory manner. However, the fact that a significant negative correlation exists at both levels of analysis indicates the stability of this correlation for different sets of samples. Moreover, the results obtained from the *NONO* KO independently verify its regulatory function in the control of *MS4A1* expression and support the biological explanation of the correlations.

Altogether, these indicate that NONO expression is linked to CD20 transcript regulation in B-ALL. A better understanding of how NONO is associated with CD20 regulation may improve mechanistic insight into antigen heterogeneity in B-ALL.

In addition to its regulatory impact on CD20 protein levels, a comprehensive analysis of the pre-mRNA splicing patterns of the *MS4A1* gene provides deeper insights into the molecular mechanisms that shape B cell identity and contribute to immune escape, particularly under pathological conditions such as lymphoid malignancies (11, 27, 30). Alternative splicing represents the primary mechanism distinguishing the two CD20 isoforms, WT- and D393-CD20 (26). In the D393-CD20 isoform, an alternative splicing pattern is employed in which a substantial portion of the sequence between exons 3 and 7 is skipped (26, 27). This variant, by missing part of the extracellular loop of CD20, can reduce the accessibility of the target epitope to anti-CD20 antibody (26). Research suggests that expression of the D393-CD20 isoform is specific to B-cell malignancies and is not seen in other diseases or benign immune conditions (27).

Henry et al. reported that the ΔCD20 (D393-CD20) transcript is detectable in various B-cell malignancies, including follicular lymphoma (FL), mantle cell lymphoma (MCL), diffuse large B-cell lymphoma (DLBCL), and B-ALL (26). In a cohort of 27 B-ALL patients, this alternatively spliced isoform accounted for an average of 3.6% of total CD20 transcripts, although this amount varies between patients (26). This percentage was lower than that observed in *in vitro*-generated B cell blasts or certain mature B-cell lymphomas, such as DLBCL in lymph nodes (26). In this regard, we investigated the D393-CD20 mRNA expression in our cohort of pediatric B-ALL blasts with variable CD20 expression, and showed that the D393-CD20 splice variant is detectable in leukemic blasts that express CD20 over 39% but it was undetectable or minimally present in CD20-negative or slightly positive blasts. In addition, although the D393-CD20 variant encodes an intracellular, non-surface protein, its transcript levels correlate positively with both WT-CD20 mRNA and surface CD20 levels. The observed positive correlations suggested a shared regulatory program co-regulating both splice isoforms. Moreover, a significant positive correlation between the D393/WT−CD20 mRNA ratio and total *MS4A1* transcript abundance (EX7−8) in B−ALL suggests a splicing bias emerging under high transcriptional load, favoring the alternative D393−CD20 isoform.

WT−CD20 overexpression elevated *MS4A1* mRNA levels while skewing splicing toward the D393−CD20 variant. This isoform enrichment was not detected using a PCR assay amplifying from exon 3 to the endogenous 3′ UTR, suggesting that the upregulated transcript originates from a non-genomic, plasmid-derived source. A WT-CD20 construct harboring a change of the third nucleotide of the acceptor site at position 612 abolished the short variant at both mRNA and protein levels (26). These findings suggest that WT-CD20 mRNA possesses an intrinsic capacity for re-splicing, operating independently of canonical intronic architecture. This reveals a previously underappreciated layer of post-transcriptional flexibility, which may become apparent under pathological conditions or in cases of transcript overabundance. The pathway appears to be driven largely by cryptic splice-sites within exons and, for CD20, likely co-opts evolutionary vestiges of exonized introns.

Splicing is a dynamic process shaped by the cellular environment (37, 38). Depending on the lineage and species-specific repertoire of splicing regulators, the identical transcript can be processed into completely different isoform patterns (39, 40). Consistent with this principle, CD20 showed strikingly divergent splicing outcomes after WT-CD20 transfection. Notably, neither 293T (HEK 293T; human embryonic kidney 293 cells) nor PG13 cells (a mouse-derived amphotropic packaging cell line) endogenously express CD20, and the reported splicing patterns were only observed after exogenous expression of a CD20 cDNA construct (26). In this context, 293T cells predominantly produced the shorter isoform, while in PG13, the WT-CD20 isoform remained dominant (26). In our study, WT-CD20 transfection of the 697 cell line (human B-cell precursor leukemia) resulted in a pronounced shift towards the short isoform at the mRNA level, together with no detectable alteration in surface CD20 protein, suggesting that hematopoietic malignancies may create a uniquely permissive environment in which dysregulated RBPs facilitate aberrant splice-site usage. Importantly, we cannot exclude that experimental variables, including plasmid dosage or transfection efficiency, also contributed to the observed variability. Whether the molecular subtype of B-ALL has an impact on the efficacy of the splicing remains to be clarified. The 697 cell line used in this work has a near diploid karyotype and expression of fusion gene *TCF3-PBX*, which has been associated to a treatment-dependent outcome, with improved prognosis by using intensification protocols (41). However, in the literature, CD20 positivity (defined as CD20 > 20%) has not be associated with molecular subtypes or sex, with the exception that *MLL-AF4* patients do not express CD20. Association was found with the age, with children between 1 and 10 years being more likely to express CD20 as compared to infants younger than 1 year of age or patients older than 10 years (7, 42). Our cohort is to small to find an association between high CD20 expression (defined as CD20 (≥39%) and molecular subgroups, however CD20 high expression was found particularly in hyperploid samples and was not found in *ETV6-RUNX1* samples. The effect of the treatment protocol on CD20 levels should be considered in future analyses, as the intensity of CD20 expression increases with the use of glucocorticoids during induction therapy (5).

Taken together, these results suggest that CD20 splicing is highly context-sensitive and likely shaped by both cryptic splice-site activation and environmental factors. Although our data are limited to a single hematopoietic cell line, the variability observed across systems hints that pathological or artificial contexts may drive abnormal isoform generation, potentially contributing to cancer-associated signatures or creating therapeutic opportunities through splicing modulation.

An increasingly recognized mechanism of therapeutic resistance in B-cell malignancies involves alternative splicing of surface antigen genes, which can lead to the loss of epitopes required for immune recognition. The findings for CD19 and CD20 suggest that although both antigens undergo splicing rearrangements under treatment-induced selection pressure, their functional consequences differ (26, 43). In the case of CD19 in human B-ALL samples, the emergence of the exon 2-skipped variant (Δex2), which lacks the CAR-binding epitope, represents a robust adaptive response to CAR-T cell pressure (43, 44).

In contrast, following rituximab treatment, expression of the full-length CD20 isoform (WT-CD20) typically remains detectable, although the truncated D393-CD20 isoform becomes enriched in resistant cell lines such as RAMOS R2–64 and RAJI R2-2 (26).

Clinical data in patients with follicular lymphoma and mantle cell lymphoma show that D393-CD20 expression increases after rituximab therapy, particularly in those with early relapse or incomplete response (26). Our molecular results suggest that the observed increase may be partly due to the re-splicing of the WT-CD20 transcript into the D393-CD20. This may increase the proportion of the truncated D393-CD20 isoform, which is not translated into a surface-expressed protein. Whether such isoform shifts directly affect antibody-mediated recognition *in vivo* remains to be determined. However, due to the relative persistence of the wild-type isoform, the resistance developed may be partial and incomplete, and the effectiveness of the treatment may not be completely lost.

Taken together, the regulation of CD20 gene expression, through both RBPs and alternative splicing, represents a key determinant of antibody therapy response. Our data suggest that D393-CD20 may arise via the resplicing of mature WT-CD20 mRNA, representing a potential mechanism contributing to altered isoform composition. Whether such alterations functionally influence antibody-mediated recognition requires further investigation. However, confirmation of the endogenous nature of this resplicing process and identification of the molecular factors that drive it will require future investigation, as their targeted inhibition could help prevent the development of escape isoforms and lower the risk of disease relapse. CD20 splicing correlates with high levels of CD20 expression. Given the association between CD20 expression, glucocorticoid exposure, and patient age, these parameters should be considered when evaluating the potential application of anti-CD20 therapies in B-ALL. , , .

## Data Availability

The original contributions presented in the study are included in the article/**Supplementary Material**. Further inquiries can be directed to the corresponding author.

## Supplementary material

Supplementary Figure 1 Validation of *NONO* knockout efficiency. Relative expression of *NONO* mRNA in control and *NONO* KO 697 cells was quantified by qRT–PCR and normalized to control (set to 100%). *NONO* transcript abundance was reduced by ~62% upon KO.

Supplementary Figure 2 Visualization of *CD20* isoforms via agarose gel electrophoresis following WT-CD20 transfection. Conventional PCR using primers spanning exons 3–8 of the *MS4A1* coding sequence revealed two isoforms following WT-CD20 transfection in 697 cells: the full-length WT-CD20 (~894 bp) and the shorter D393-CD20 variant (~393 bp). PCR products were resolved on a 1% agarose gel and visualized by Midori Green staining, shown in two independent experiments (#Exp.1 and #Exp.2).
