# Supplementary figures and images for: Dual-trigger model of CD20 escape: NONO regulation and cryptic splicing induced by transcript overload in pediatric B-ALL
Source: Front Immunol. 2026 Apr 1;17:1763413. doi: 10.3389/fimmu.2026.1763413 (PMC13079139; doi:10.3389/fimmu.2026.1763413)

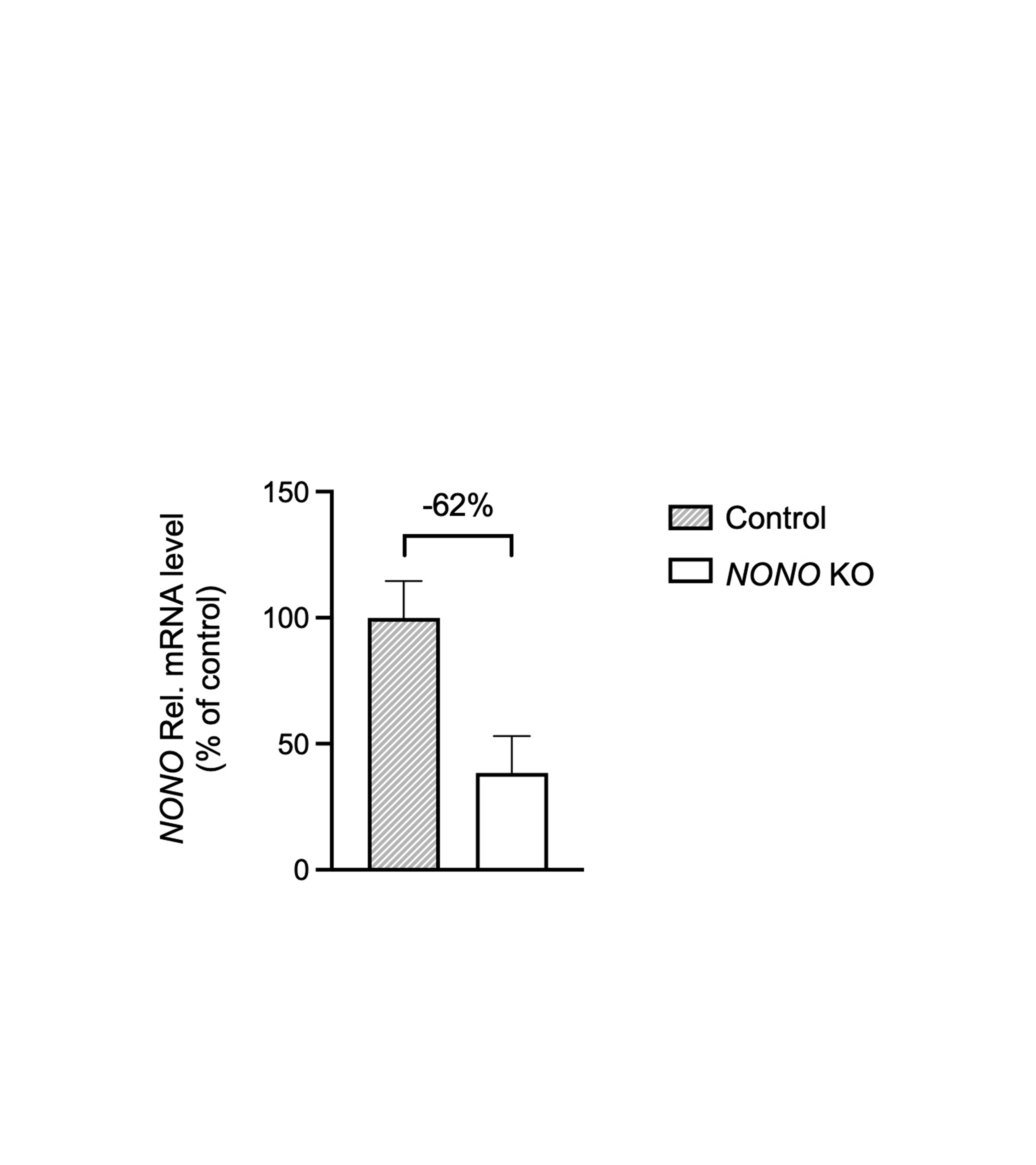

Supplement: Supplementary Figure 1 — Validation of NONO knockout efficiency. Relative expression of NONO mRNA in control and NONO KO 697 cells was quantified by qRT–PCR and normalized to control (set to 100%). NONO transcript abundance was reduced by ~62% upon KO. [file Image1.tif]

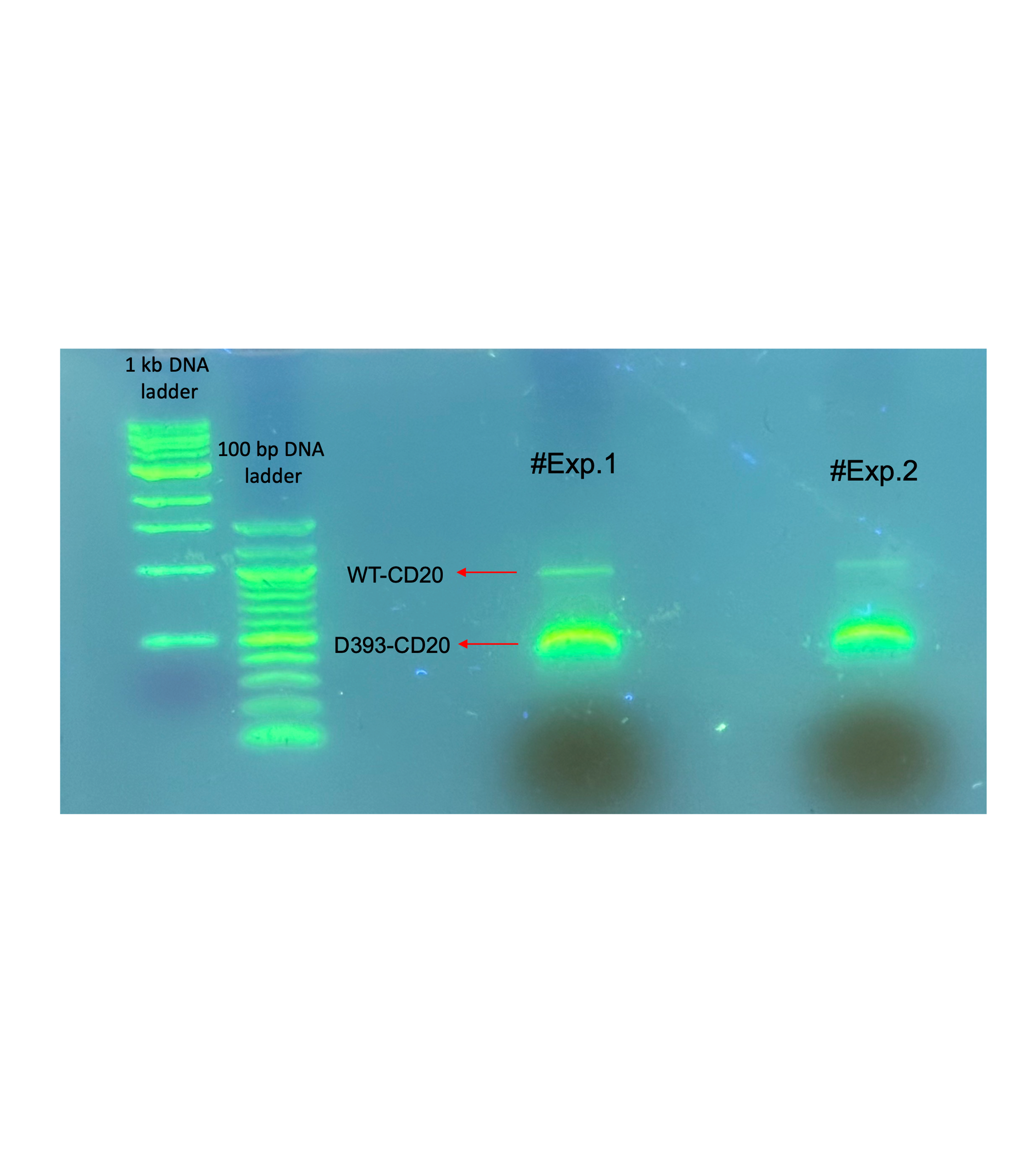

Supplement: Supplementary Figure 2 — Visualization of CD20 isoforms via agarose gel electrophoresis following WT-CD20 transfection. Conventional PCR using primers spanning exons 3–8 of the MS4A1 coding sequence revealed two isoforms following WT-CD20 transfection in 697 cells: the full-length WT-CD20 (~894 bp) and the shorter D393-CD20 variant (~393 bp). PCR products were resolved on a 1% agarose gel and visualized by Midori Green staining, shown in two independent experiments (#Exp.1 and #Exp.2). [file Image2.tif]
